# Healthcare costs and outcomes associated with surgical site infections after coronary artery bypass grafting surgeries in Oman

**DOI:** 10.1080/07853890.2023.2184486

**Published:** 2023-03-01

**Authors:** Fatma M. AlRiyami, Omar M. Al-Rawajfah, Sulaiman Al Sabei, Hilal A. Al Sabti, Atika Khalaf

**Affiliations:** a Cardiothoracic Unit, Sultan Qaboos University Hospital, Muscat, Oman; b Faculty of Nursing, Al Al-Bayt University, Mafraq, Jordan; c College of Nursing, Sultan Qaboos University, Muscat, Oman; d Faculty of Health Sciences, Kristianstad University, Kristianstad, Sweden; e Department of Nursing, Fatima College of Health Sciences, Ajman, United Arab Emirates

**Keywords:** Surgical site infection, coronary bypass graft surgeries, healthcare outcomes, healthcare costs

## Abstract

**Background:**

Surgical site infection (SSI) after coronary artery bypass graft (CABG) surgeries is considered a key indicator of the quality of healthcare services.

**Objective:**

This study aimed to estimate the healthcare outcomes associated with SSIs after CABG surgeries in Oman in terms of mortality rate, case-fatality rate, LOS, readmission rate and healthcare costs.

**Methods:**

The nested case-control study design was used based on retrospective data, which was conducted from 2016 to 2017. The case group encompassed all CABG patients with confirmed SSIs within 30 days of the surgery (*n* = 104) while controls were CABG patients without SSIs (*n* = 404).

**Results:**

Forty-four (42.3%) of the SSI patients were readmitted to the hospital compared to eight (2%) of the control group (*p* < .001). Patients in the case group had a longer LOS (*M =* 24.4, *SD* = 44.6 days) compared to those in the control group (*M* = 11, *SD* = 21 days, *p =* .003). The mean healthcare costs of cases (*M* = Omani Rial [OMR] 3823, *SD* = OMR 2516) were significantly greater than controls (*M* = OMR 3154, *SD* = OMR 1415, *p* = .010).

**Conclusion:**

Results from this study can be baseline data for formulating new hypotheses and testing the causal relationship between SSIs after CABG surgeries and the readmission rate, LOS and health care costs.

**Key messages**
- Surgical Site Infections (SSIs) are still a major complication after cardiac surgeries in Oman.
- SSIs after cardiac surgeries are associated with substantially increased healthcare costs and length of stay.
- SSIs after cardiac surgeries are associated with negative outcomes such as mortality and case-fatality rates.

## Introduction

More than 20% of hospital-acquired infections are attributed to surgical site infection (SSI) [1]. In the United States, SSIs occur in approximately 1% to 3% of 100 coronary bypass graft (CABG) surgeries [2]. The European Center for Disease Prevention and Control (CDC) estimated an overall SSIs rate of 2.6% after CBAG surgeries [3]. In developing countries, the rate can reach up to 23.3% [4]. The most recent study in Oman reported an infection rate of 17.5% [5].

A growing body of literature found that SSIs after CABG surgeries are associated with increased readmission, mortality and case-fatality rates [6,7]. Furthermore, previous studies have shown that SSIs after CABG surgery have resulted in a significant increase in length of stay (LOS) and healthcare cost [8,9]. In the USA, the readmission rate after CABG surgery due to SSIs ranged from 16.9% [10], to 34% [2]. Likewise, in Germany, the readmission rate due to SSIs reached 33% [7]. The problem of SSIs is not only associated with a high readmission rate but also increases the LOS. In the USA, in a large-scale study [9], SSIs resulted in about 11.8 extra LOS days after CABG surgeries. Likewise in Jordan [11] reported that SSIs are associated with an increase of the LOS by about nine days after CABG surgeries.

The available shreds of evidence suggested that the healthcare costs associated with SSIs are increasing as the readmission rate and LOS are increased. For example, in the USA, it was estimated that SSIs added $19,222 in costs for CABG patients [9]. In another study in the USA, the figure increased to $33,318 for isolated CABG patients [8]. Regionally, in a Jordanian study, SSIs added about $3159 to the healthcare cost of CABG surgery [11].

The negative outcomes associated with SSIs after CABG surgery are not only limited to prolonged LOS and increased healthcare costs, but also contributed to the mortality rate. In the UK, the reported case fatality for patients with the SSIs after CABG surgery was 9.1% compared with a 2.6% mortality rate in patients without SSIs [6]. Similarly, in Germany, the case-fatality rate reached 16.95% [7].

The standard goal of a healthcare organization is to balance quality of care and cost-effectiveness [12]. From this view, there is great importance to estimate the healthcare costs of SSIs after CABG surgeries. This estimation is a key factor in raising awareness among clinicians and healthcare policymakers about the impact of this preventable complication. Furthermore, estimating the healthcare costs of SSIs after CABG surgeries will guide healthcare policymakers and clinicians to optimize the resources utilization and develop healthcare strategic plans at the hospital and national levels. In Oman, CABG has been a standard procedure since 1999 [13], but no previous studies have been conducted to document health-related outcomes of SSIs after the surgery. The current study is a continuation of a previous published work. In our previous study [5], incidence and risk factors of SSIs after CABG surgeries in Oman were investigated. In the current study, the healthcare outcomes related to the SSIs after CABG were investigated. Therefore, the purpose of this study was to investigate the outcomes associated with SSIs after CABG surgeries in Oman in terms of mortality rate, case-fatality rate, LOS, readmission rate and healthcare costs.

## Materials and methods

### Study design

We implemented the nested case-control study design based on retrospective existing data, which was conducted from 2016 to 2017. All CABG patients within the study period constituted the study population. The data were collected from the two referral hospitals in Oman where all CABG surgeries are performed. Therefore, data from this study are generalizable to CABG surgeries in Oman. The inclusion criteria applied in this study were:

- Omani nationals >18 years old patients;
- Underwent CABG surgeries between 2016 and 2017 in the study hospitals. Patients who underwent CABG surgery more than once during the study period were counted as new case for each surgery; and
- Had electronic medical record in the study hospitals.

We excluded non-Omani patients because there were a few patients (*N* = 17), and non-Omani patients are mostly self-sponsored. This factor can significantly affect the treatment options and LOS. To avoid possible bias that may be associated with this group, we excluded them from the study. The cases group encompassed all CABG patients with confirmed SSIs within 30 days of the surgery. On the other hand, controls were CABG patients free from SSI within the study period.

### Participants

**Figure 1.**
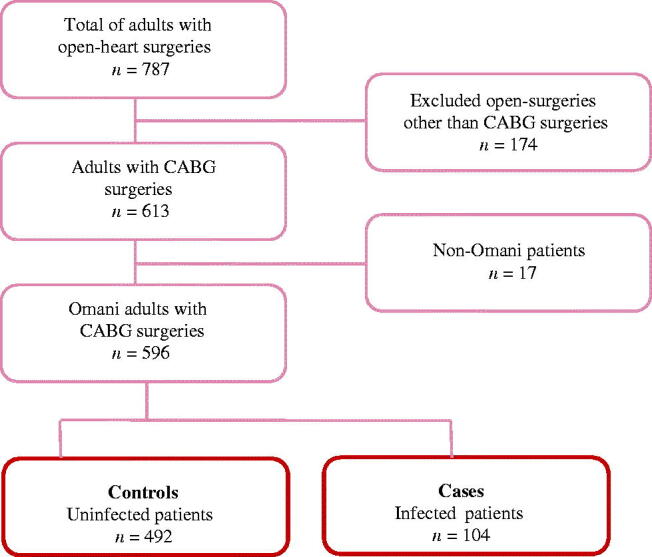
Cases and controls finding methods.

This study used consensus review and simple random sampling techniques. A consensus review was used to review all CABG patients’ (*n* = 596) medical records to identify the case group (infected patients, *n* = 104). The SSI cases were screened against the standard CDC definition of SSI [14]. Therefore, the patient’s medical records were reviewed for evidence of SSI including the laboratory reports, wound care notes and physician and nursing notes. On the other hand, simple random sampling was used to select the control group from the uninfected patients based on a ratio of 1:4. Therefore, the final control group was composed of 404 uninfected CABG patients (Figure 1). The 1:4 ratio was selected for the current study based on the recommendation of enrolling more than one control for every case [15] and previous epidemiological studies recommended using a ratio of 1:4 to achieve acceptable power [16].

### Outcome variables

In the current study, the following constitute the outcome variables:

#### Mortality rate

Constitutes the proportion of the CABG patients who died during the study period to the total study population (all CABG patients).

#### Case fatality rate

Constitutes the proportion of the CABG patients with SSIs who died during the study period to the total SSI patients.

#### Readmission rate

The proportion of the patients who were readmitted to the hospital within 30 days after the surgery because of SSI complication. Although all cases were readmitted because of SSI, other possible reasons that contributed to the readmission were not specified in this study. Reasons for readmission of controls were not specified either.

#### Length of stay (LOS)

The mean difference of the total hospital LOS between the cases and controls. The LOS of each readmission episode is added to the total extra LOS.

#### Healthcare cost

The healthcare cost associated with SSIs was calculated based on the mean difference of the total charges of the patients in the cases and controls groups. The total charges were retrieved from the healthcare information system of the participating hospitals. The retrieved data were the total charges per each patient’s admission. Detailed components of the patient’s bill were not available. Therefore, the total charge in these bills represents the charges for the LOS in the hospital, besides other unspecified healthcare charges for each patient’s admission.

### Data collection

The data were collected based on a data collection sheet that was designed for this study. The sheet was developed based on a critical review of the literature on possible risk factors and outcomes of SSI. The content of the sheet was validated by two PhD researchers: one cardiothoracic surgeon and one infectious disease consultant.

### Data analysis

Data were entered into the data analysis software (IBM Corp. Released 2015. IBM SPSS Statistics for Windows, Version 23.0. Armonk, NY: IBM Corp.). A random sample of 5% of the data collection sheets was selected and then crosschecks with data entered in the SPSS data file. Variables with >10% missing data were excluded from the final analysis. This study utilized descriptive and inferential statistics. Descriptive analyses such as frequency, percentage, mean and standard deviation were used to describe the study sample. Chi-square and independent *t*-test were used to test the significant differences in different variables between the case and control groups.

### Research ethics

The study protocol was ethically approved by the ethical review board in the university of the principal investigator, Sultan Qaboos University (Protocol approval Code: No. SQU-EC/092/19) and the Ministry of Health in Oman (Approval #1902). In addition, this work was carried out in accordance with the guidelines and recommendations of the Declaration of Helsinki.

## Results

A total of 104 SSIs were identified during the study period, which reveals an overall incidence rate of 17.45 cases per 100 CABG surgeries. More details about the incidence calculation, causative microorganisms and risk factors were presented in a previous publication [5]. According to the CDC classification of the SSIs, 59.6% (*n* = 62) were superficial infections and the rest 40.4% (*n* = 42) were deep tissue infections. The mean age for cases was 62.3 years (*SD* = 8.6) compared to 63.8 years (*SD* = 9.2) for controls. For the majority of the patients (88.7%, *n* = 447) both mammary artery and saphenous vein were used for the graft. Whereas in 3% (*n* = 15) of the patients, only the mammary artery was used. The majority of the patients (59.6% of cases and 80.9% of controls) were male. About 86.5% of the cases were diabetic compared to 49.1% of the control group (Table 1).

**Table 1.** Sample characteristics (*N* = 508).

Variables	Cases	Controls	*χ* ^2^	*p*
	(*N* = 104)	(*N* = 404)		
	*N* (%)	*N* (%)		
Mean age (*SD*)	62.3 (8.6)	63.8 (9.2)	*t* = 1.5	.130
Sex				
Male	62 (59.6)	327 (80.9)	21	<.010
Female	42 (40.4)	77 (19.1)		
Body mass index^a^				
Obese	39 (38.2)	100 (25.4)	18	<.010
Overweight	45 (44.1)	137 (34.8)		
Normal weight	17 (16.7)	147 (37.3)		
Under weight	1 (1)	10 (2.5)		
Type of surgery				
Elective	91 (87.5)	381 (94.3)	6	.030
Emergency	13 (12.5)	23 (5.7)		
Discharge status				
Discharge home	98 (94.2)	379 (93.8)	0.52	.770
Died in hospital	6 (5.8)	23 (5.7)		
Transfer to other hospital	0 (0)	2 (0.5)		
Infection site for cases				
Sternal wound	64 (61.5)	–		
Leg wound	34 (32.7)	–		
Sternal and leg wounds	6 (5.8)	–		
Wound classification				
Superficial wound infection	62 (59.6)	–		
Deep wound infection	42 (40.4)	–		
Wound management^b^				
Dressing	79 (77.5)	–		
Wound debridement under general anaesthesia	9 (8.8)			
Vacuum assisted device	14 (13.7)			

^a^Missing data in the control group: 10 cases for body mass index. Missing data in the case group: two cases each for body mass index.

^b^Missing data in the case group: two cases for the wound management.

In this study, the outcome variables for SSIs after CABG surgeries were mortality rate, case fatality rate, readmission rate, LOS and healthcare costs. Out of the total SSI patients, 44 (42.3%) patients were readmitted to the hospital compared to 8 (2%) from controls (*p* < .001). The overall crude mortality rate was 3%. Furthermore, six of SSI patients died in the hospital, which reveals a 5.8% case fatality rate. Furthermore, patients with SSIs stayed significantly longer in the hospitals (*M =* 24.4, *SD* = 44.6 days) compared to uninfected patients (*M* = 11, *SD* = 21 days, *p =* .003). The mean healthcare costs of patients with SSIs (*M* = Omani Rial [OMR] 3823, *SD* = OMR 2516) was significantly greater compared to non-infected patients (*M* = OMR 3154, *SD* = OMR 1415, *p* = .010) (Table 2).

**Table 2.** Comparison of different health-related outcomes for case and control groups.

Variables	Cases	Controls	*t* (*df*)	*p*
	*N* = 104	*N* = 404		
	*M* (*SD)*	*M* (*SD*)		
Length of stay	24.4 (44.6)	11 (21.0)	4.4 (501)	.003
Healthcare costs^a^	3823 (2516)	3154 (1415)	2.6 (506)	.010
	*n* (%)	*n* (%)	*χ*^2^ (df)	*p*
Readmission rate	44 (42.3)	8 (2)	146.4 (1)	<.001
Fatality rate	6 (5.8)	23 (5.7)	0.001 (1)	1.000

^a^Healthcare cost in Omani Rial OMR (1 OMR = 2.60 USD).

## Discussion

Similar to other types of healthcare-associated infections [17], the results from this study revealed that the incidence rate of SSIs after CABG surgeries in Oman is high compared to the reported rates from the developing countries. Comparison and discussion of this rate are presented in a previous publication [5]. In this study, the calculated crude mortality rate was 3%, which is within the range of the reported mortality rates in other studies [18,19]. Several factors may cause death for the CABG patients, such as myocardial infarction [20,21], heart failure [22], bleeding [23,24] or stroke [25]. Although the current study reported the crude mortality rate, it did not report the associated causes of death. Future studies may consider reporting the associated causes of death after CABG surgeries.

The case fatality rate in this study was 5.8%. This rate is lower compared to previous studies, which reported a fatality rate ranging from 8.4% [26] to 16.9% [7]. Although the calculated case fatality rate was based on a standard equation, the cause of the death may be related to something other than SSIs. Therefore, the reported figure should be interpreted by considering that this study was based on retrospective data in which the direct cause of death may be difficult to identify.

The readmission rate in this study was 44.2% for infected patients compared to 2.2% for uninfected patients. This readmission rate is higher compared to previous studies [2,10]. This discrepancy may be explained because the hospitals in the current study are the only centres performing CABG surgeries in Oman. Any complications after the CABG surgery mostly necessitate that patients must return to the hospital where they had the surgery. Finally, although the readmission of the infected group was due to SSI, they might be readmitted because of the existence of other surgical complications at the same time. Future research could give more insight into the causes of readmission among CABG patients in Oman.

The current study showed that patients with SSIs after CABG surgeries had an additional 13 days added to their LOS compared to uninfected patients. This result is consistent with previous studies [2,8,9]. An increase in LOS after developing SSI is expected due to the treatment course for managing the SSI; the need for antibiotic therapy or the need for an additional surgical procedure [27]. In addition, using negative pressure therapy to promote blood flow stimulates granulation tissue. This technique stimulates cell proliferation and wound healing but requires an average of 30 days to promote wound healing [28]. Hence, the patient’s LOS will be extended.

The current study is probably the first one from Oman to estimate the healthcare costs associated with SSIs after CABG surgeries. We estimated that, on average, SSI added OMR 669 to the patients’ healthcare costs. These extra healthcare costs are less than those reported in other studies [8,9]. The results from this study should be interpreted taking into consideration that there is no well-developed billing system in the Omani governmental hospitals, where the study was conducted. All Omani patients are covered by governmental universal insurance, which provides medical services free of charge. Notably, the current reported healthcare costs do not include medical and nursing procedures related to SSI management (e.g. wound dressing, dressing sets, sterile and non-sterile gloves, gauze, cleaning solutions, applying vacuum assistive devices, etc.), medical consultation, medical and surgical supplies and equipment used during the hospital stay. Therefore, the reported increase in healthcare costs most likely underestimates the actual healthcare costs associated with SSIs. Future studies may use other approaches to include healthcare cost components that are not considered in this study.

### Limitations

Finally, although this study is probably the first Omani study to report health-related outcomes of SSI after CABG surgeries, a few limitations should be mentioned. This study relied on retrospective data, which resulted in some paucity in the collected data, such as the reason for readmission in control group and accurate cause of deaths. Therefore, future studies may consider prospective designs to fill these paucities. Furthermore, the generated figures related to the healthcare cost might not accurately reflect the extra cost burden associated with SSI after CABG surgeries. These figures were generated based on the currently available billing system. Therefore, these figures should be interpreted by taking into consideration the paucity of the billing system.

## Conclusions

The results from the current study can be used to inform all healthcare stakeholders in Oman and similar countries, including clinicians, educationists, researchers and healthcare policymakers on the importance of and the need for planning of interventions to combat the current high prevalence of SSIs after CABG surgeries. For healthcare professionals, the results from this study may increase awareness of the impact of SSIs after CABG surgery on the healthcare system in terms of mortality rate, LOS, readmission rate and healthcare costs. Finally, the results from the current study can be baseline data for formulating new hypotheses and test the causal relationship between SSIs after CABG surgeries and the readmission rate, LOS and health care costs.

## Data Availability

The datasets analysed during the current study are available from the first author [Fatma AlRiyami] on reasonable request.
